# Postoperative delirium is an independent factor influencing the length of stay of elderly patients in the intensive care unit and in hospital

**DOI:** 10.1007/s00540-022-03049-4

**Published:** 2022-02-19

**Authors:** Andrea Kirfel, Vera Guttenthaler, Andreas Mayr, Mark Coburn, Jan Menzenbach, Maria Wittmann

**Affiliations:** 1 Department of Anesthesiology and Intensive Care Medicine, University Hospital Bonn, Venusberg-Campus 1, 53127 Bonn, Germany; 2 Institute for Medical Biometry, Informatics and Epidemiology, University Hospital Bonn, Venusberg-Campus 1, 53127 Bonn, Germany

**Keywords:** Postoperative delirium, Elderly patients, Length of stay

## Abstract

**Purpose:**

Postoperative delirium (POD) is an often unrecognized adverse event in older people after surgery. The aim of this subgroup analysis of the PRe-Operative Prediction of postoperative DElirium by appropriate SCreening (PROPDESC) trial in patients aged 70 years and older was to identify preoperative risk factors and the impact of POD on length of stay (LOS) in intensive care unit (ICU) and hospital.

**Methods:**

Of the total 1097 patients recruited at a German university hospital (from September 2018 to October 2019) in the PROPDESC prospective observational study, 588 patients aged 70 years and older (mean age 77.2 ± 4.7 years) were included for subgroup analysis. The primary endpoint POD was considered positive if one of the following tests were positive on any of the five postoperative visit days: Confusion Assessment Method for ICU (CAM-ICU), Confusion Assessment Method (CAM), 4'A's (4AT) and Delirium Observation Scale (DOS). Trained doctoral students carried out these visitations and additionally the nursing staff were interviewed for completion of the DOS. To evaluate the independent effect of POD on LOS in ICU and in hospital, a multi-variable linear regression analysis was performed.

**Results:**

The POD incidence was 25.9%. The results of our model showed POD as an independent predictor for a prolonged LOS in ICU (36%; 95% CI 4–78%; < 0.001) and in hospital (22%; 95% CI 4–43%; < 0.001).

**Conclusion:**

POD has an independent impact on LOS in ICU and in hospital. Based on the effect of POD for the elderly, a standardized risk screening is required.

**Trail registration:**

German Registry for Clinical Studies: DRKS00015715.

## Introduction

The older generation will continue to grow steadily in the coming years. In 2050, the number of people aged 70 and older will nearly have doubled from 5.9 to 11.3% [1]. With increasing age, people suffer more frequently from diseases and often develop multimorbidity [2]. Additionally, the incidence of cognitive impairment in the elderly is also not to be underestimated. Severity of illness, cognitive impairment, as well as functional, visual, and hearing impairment, are considered risk factors for postoperative delirium in literature [3–8].

Postoperative delirium (POD) is an often unrecognized postoperative adverse event in the elderly [3, 9–11]. Defined by the Diagnostic and Statistical Manual of Mental Disorders, Fifth Edition (DSM-5) and the 10th revision of the International Statistical Classification of Disease and Related Health Problems (ICD-10), delirium is an acute and fluctuating disturbance of awareness, attention and cognition caused by an organic pathophysiology [12, 13]. In the literature, the clinical presentation of POD is divided into hypoactive, hyperactive and mixed forms. Whereas the occurrence of hypoactive delirium is often underestimated in everyday clinical practice, hyperactive delirium makes patient´s care very time-consuming [14–16].

The incidence of POD varies in different surgical populations from 11 to 51% [3, 9, 10]. In addition to various outcome deteriorations such as cognitive impairment and other postoperative complications, the effects of POD on the length of stay (LOS) are also often reported [17–20]. Regardless of the complication of POD, prolonged length of stay is often mentioned as an cost-increasing factor in various studies of patients who underwent surgery [21].

In conjunction with a prolonged hospital stay, elderly patients, in particular, may experience additional loss of function that can severely impact the ability to continue an independent life [22]. However, the prolonged stay and complication of POD is not only a burden for patients, but also for nurses [23]. Furthermore, it is commonly known that there is a shortage of nurses and physicians in hospitals. Thus, there is a limited human resource for high-quality care of elderly patients. For all intents and purposes, this means that a prolonged length of stay in combination with a POD puts a strain on the limited resources and makes it even more difficult to provide needs-based care for older affected people [24]. POD is a postoperative complication influenced by various perioperative risk factors, which can be counteracted protectively [5].

This subgroup analysis was performed to figure out the risk factors for POD (age, surrogate parameters for multimorbidity, surgery associated risk factors) in the elderly in more detail and to take a closer look at the effects of these factors on the length of stay. Further, this analysis will examine whether POD is an independent risk factor for a prolonged stay in an intensive care unit (ICU) and in hospital.

## Materials and methods

### Study design and participants

This is a subgroup analysis of an observational prospective single-centre trial on patients from different surgical disciplines of the University Hospital Bonn. The entire study, conducted from September 2018 to October 2019 under the title "PRe-Operative Prediction of postoperative DElirium by appropriate SCreening (PROPDESC)" included 1097 patients [25]. It was registered in the German Registry for Clinical Studies under the number DRKS00015715 and was approved by the local institutional Ethics Committee at the Medical Faculty of the Rheinische Friedrich-Wilhelms-University of Bonn. Written informed consent was obtained from each patient. Patients with age 60 and older and with a planned surgery duration of at least 60 min were eligible for the PROPDESC study. Exclusion criteria were emergency procedures, language barriers or missing compliance with the study protocol.

The subgroup analyzed here included all enrolled patients aged 70 and older. The patient data pertain to the inpatient period and the discharge date.

### Data collection

In this subgroup analysis, 15 variables were included. Preoperative data collected include the following: age, sex, body-mass-index (BMI), cognitive impairment tested with the Montreal Cognitive Assessment (MoCA), hearing impairment (yes or no), POD in the medical history (yes or no), the number of long-term medication, American Society of Anesthesiologists (ASA) Physical Status Classification System, Revised Cardiac Risk Index (rCRI), New York Heart Association Classification (NYHA), Metabolic Equivalent of Tasks (MET), surgical risk and surgical discipline. Surgical risk was transformed from a 5-level Johns-Hopkins classification to the 3-level modified Johns-Hopkins surgical criteria [26, 27]. Intraoperative data collected include red blood cell transfusion and ventilation time. Postoperative data collected include surgery duration, length of stay (LOS) in the intensive care unit (ICU) and LOS in hospital.

### Patient outcome

The primary endpoint of POD was assessed on the first five consecutive days after surgery, alternatively after the end of sedation. Sedated patients with RASS [28] score < − 3 were considered as not assessable and therefore their testing for POD was initiated after exceeding this level of sedation according to Confusion Assessment Method for ICU (CAM-ICU) [29].

Trained doctoral students performed the testing. In order not to miss a positive POD diagnosis, different tests were applied in the PROPDESC study. CAM-ICU was used for intensive care patients and Confusion Assessment Method (CAM) and the 4 ‘A’s (4AT) were conducted in patients on the normal ward [29–31]. To avoid missing delirium diagnosis in the context of spot examinations, the Delirium Observation Scale (DOS) was additionally applied by interviewing the nursing staff to assess the previous 24 h [32]. The positive endpoint POD was considered if one of the applied delirium assessments was positive on at least one visit day. The definition of completed POD assessment required a valid conduct of at least three of the five scheduled postoperative visits. Discharge home before the third visit was accepted as an exception to this rule, on the assumption that patients would not subsequently become delirious in their familiar environment.

### Statistical analysis

Statistical analysis was performed using the statistical programming environment R. Continuous and ordinal variables are presented with mean and standard deviation (SD±). Nominal variables are displayed as numbers and percentages. Patients were divided into two groups (non-POD vs. POD group) based on the POD endpoint. The difference between these groups regarding the characteristics was analyzed using the non-parametric Wilcoxon rank-sum test for continuous variables. For categorical variables, Fisher´s exact test was computed to check for independence.

To evaluate the independent effect of POD on LOS in ICU and in hospital, a multi-variable linear regression analysis was performed to adjust for various perioperative potential confounders. The LOS outcomes were log-transformed to ensure approximate normality of residuals. POD was entered as a binary variable while adjusting for perioperative risk factors for POD. These covariates were preoperative age, ASA, NYHA, MET, rCRI classification levels, MoCA sum score, hearing impairment, history of delirium, number of medication and intra-/postoperative surgical risk, surgical discipline, duration of surgery, red cell blood transfusion and ventilation time. In conjunction with the multivariable linear regression analysis related to the effect of POD on LOS in ICU, only patients in the cohort who actually had an ICU stay were included and ventilation time was removed in the risk adjustment because it contains part of the outcome parameter LOS in ICU. To ensure the interpretability, the coefficients of POD from modelling the log LOS were re-transformed and are presented in the percent increase (compared to non-POD) with a corresponding 95% confidence interval. For sensitivity reasons, the regression analyses were repeated also without covariates, which contained more than 5% missing values to check for potential biases induced due to the missing observations.

## Results

**Fig. 1:**
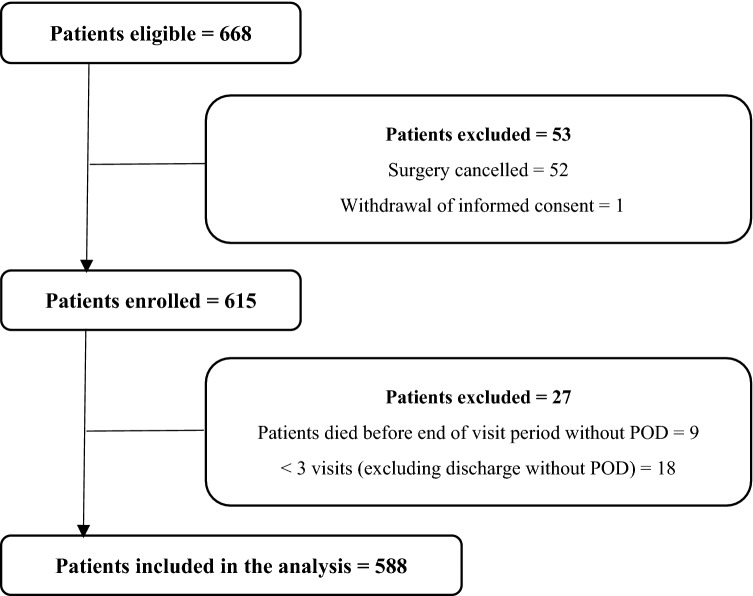
Flow chart

The subgroup of patients aged ≥ 70 years included 668 patients. Of this cohort, 52 (7.8%) patients had no surgery and one (0.1%) has withdrawn the consent during the observation period. Of the 615 patients enrolled, an additional nine (1.3%) died within the postoperative visitation period without reaching the positive endpoint of POD. Since the complete assessment of the primary endpoint of POD was not possible, these patients were also removed from the dataset. Furthermore, 18 (2.7%) patients had less than three visits completed before postoperative day 5 without having been discharged from the hospital. These patients were also removed from the evaluation cohort, and thus 588 patients were included in the analyses presented here. The flow chart (Fig. 1.) shows the case number of participants and their exclusion criteria.

### Characteristics of perioperative variables related to POD

The mean age of the subgroup analyzed here was 77.2 (± 4.7) years and the gender distribution was 248 (42.2%) women and 340 (57.8%) men. POD incidence was 25.9% (152). Table 1 shows the variables collected preoperatively and postoperatively divided into the non-POD and POD groups. Anesthetic classifications (ASA, *p* < 0.001; NYHA, *p* < 0.001; MET, *p* = 0.009 and rCRI, *p* < 0.001) were significantly different between the delirious and non-delirious groups. Furthermore, POD patients (21.4 ± 4.1) showed a significantly lower MoCA sum score than non-POD patients (22.8 ± 3.7; *p* < 0.001). The surgical risk of the cohort was significantly higher in patients who developed POD postoperatively. As expected the highest POD incidence occurred in patients after cardiac surgery with 52.6% (80). Postoperative variables also differed significantly between the delirious and non-delirious groups. POD patients had an average of 78 min longer surgery duration (non-POD 188.9 ± 115.1; POD 266.8 ± 121.9; *p* < 0.001). Furthermore, the duration of ventilation differed by about 25 h. In the non-delirious patients, the mean value of ventilation time was 7 h (7.44 ± 13.4) and in the delirious patients about 32 h (32.2 ± 24.8; *p* < 0.001). The length of intensive care stay differed by an average of 138 h between delirious (21.4 ± 62.4) and non-delirious patients (159.3 ± 525.4; *p* < 0.001). This makes a difference in the LOS in ICU of about 6 days. Also shown was a significant difference in total LOS in the hospital of about 8 days. Patients who developed POD during the visit period stayed about 26 days (25.6 ± 17.2) and patients without POD stayed an average of 17 days (17.2 ± 25.7; *p* < 0.001).

**Table 1:** Pre- and postoperative variables for the non-POD and POD group

Characteristics	Total	Non-POD	POD	*p* value	Missing values
No	588	436 (74.2)	152 (25.9)	–	–
Age	77.2 ± 4.7	77.1 ± 4.8	77.5 ± 4.6	0.245	0
*Sex (no., %)*				0.013	0
Female	248 (42.2)	197 (45.2)	51 (33.6)		
Male	340 (57.8)	239 (54.8)	101 (66.5)		
BMI	27.0 ± 4.9	27.0 ± 5.0	27.0 ± 4.6	0.688	1
No. of medication	6.0 ± 3.7	5.8 ± 3.7	6.6 ± 3.6	0.011	10
*Hearing impairment*				0.354	0
Yes	175 (29.8)	125 (28.7)	50 (32.9)		
No	413 (70.2)	311 (71.3)	102 (67.1)		
*History of POD*				0.858	1
Yes	44 (7.5)	32 (7.3)	12 (7.9)		
No	543 (92.4)	403 (92.4)	140 (92.1)		
MoCA sum	22.4 ± 3.8	22.8 ± 3.6	21.4 ± 4.1	< 0.001	0
*ASA (no., %)*				< 0.001	0
ASA 1	9 (1.5)	7 (1.6)	2 (1.3)		
ASA 2	190 (32.3)	168 (38.5)	22 (14.5)		
ASA 3	344 (58.5)	238 (54.6)	106 (69.7)		
ASA 4	45 (7.7)	23 (5.3)	22 (14.5)		
*rCRI (no., %)*				< 0.001	0
rCRI 1	237 (40.3)	207 (47.5)	30 (19.7)		
rCRI 2	141 (24.0)	105 (24.1)	36 (23.7)		
rCRI 3	146 (24.8)	94 (21.6)	52 (34.2)		
rCRI 4	64 (10.9)	30 (6.9)	34 (22.4)		
*NYHA (no., %)*				< 0.001	0
NYHA I	235 (40.0)	199 (45.6)	36 (23.7)		
NYHA II	203 (34.5)	152 (34.9)	51 (33.6)		
NYHA III	137 (23.3)	78 (17.9)	59 (38.8)		
NYHA IV	13 (2.2)	7 (1.6)	6 (4.0)		
*MET (no., %)*				0.009	0
MET< 1	9 (1.5)	7 (1.6)	2 (1.3)		
MET 1–4	307 (52.2)	210 (48.2)	97 (63.8)		
MET 5–10	255 (43.4)	205 (47.0)	50 (32.9)		
MET > 10	17 (2.9)	14 (3.2)	3 (2.0)		
*Surgical discipline (no., %)*				< 0.001	0
Cardiac surgery	152 (25.9)	72 (16.5)	80 (52.6)		
Thoracic surgery	14 (2.4)	11 (2.5)	3 (2.0)		
Abdominal surgery	65 (11.1)	56 (12.8)	9 (5.9)		
Vascular surgery	22 (3.7)	15 (3.4)	7 (4.6)		
Orthopedic surgery	222 (37.8)	187 (42.9)	35 (23.0)		
Others	113 (19.2)	95 (21.8)	18 (11.8)		
*Surgical risk (no., %)*				< 0.001	0
Low	83 (14.1)	80 (18.4)	3 (2.0)		
Intermediate	263 (44.7)	206 (47.3)	57 (37.5)		
High	242 (41.2)	150 (34.4)	92 (60.5)		
Surgery duration (min.)	209.0 ± 121.7	188.9 ± 115.1	266.8 ± 121.9	< 0.001	0
Red blood cell transfusion (ml)	420.3 ± 1509.4	221.8 ± 575.5	972.4 ± 2705.7	< 0.001	66
Ventilation time (h)	13.9 ± 65.8	7.4 ± 13.4	32.2 ± 125.5	< 0.001	7
LOS in ICU (h)	56.8 ± 277.6	21.4 ± 62.4	159.3 ± 525.4	< 0.001	8
LOS in hospital (days)	19.3 ± 25.7	17.2 ± 25.7	25.6 ± 24.8	< 0.001	11

Data are mean (±) unless stated otherwise

*POD* postoperative delirium, *BMI* body mass index, *MoCA* Montreal Cognitive Assessment, *ASA* American Society of Anesthesiology, *NYHA* New York Heart Association, *rCRI* Revised Cardiac Risk Index, *MET* metabolic equivalent of tasks, *LOS* length of stay, *ICU* Intensive Care Unit

### Characteristics of perioperative variables related to a postoperative ICU stay

To obtain a more accurate overview of the cohort that was postoperatively in intensive care, the subgroup was divided into two groups (non-ICU and ICU). Table 2 compares the intraoperative variables and the postoperative POD-Outcome for the ICU (267; 45.4%) and non-ICU (313; 53.2%) group. The average stay of intensive care patients was 123.4 h (± 399.4). Fifty-five point four percent (148) of the patients on ICU underwent cardiac surgery and 71.5% (191) had a high-risk surgery. Patients with a postoperative intensive care stay showed an average of 149 min longer surgery duration (non-ICU 140.9 ± 77.6 min; ICU 290.1 ± 114.9 min; *p* < 0.001). Furthermore, the two groups differed significantly in red blood cell transfusion (non-ICU 51.1 ± 277.4 ml; ICU 856.0 ± 2136.6 ml; *p* < 0.001) and ventilation time (non-ICU 3.6 ± 1.5 h; ICU 26.0 ± 95.7 h; *p* < 0.001). The non-ICU and ICU groups also differed significantly in postoperative outcomes related to POD and overall LOS in hospital. Forty-two point three percent (113) of intensive care patients developed POD and only 11.5% (36) of non-intensive care patients were tested positive. Patients with ICU stay showed an average of 9 days longer total hospital stay (non-ICU 15.4 ± 20.6 days; ICU 24.0 ± 30.3 days; *p* < 0.001).

**Table 2:** Pre- and postoperative variables for the non-ICU and ICU stay

Characteristics	Non-ICU	ICU	*p* value
No	313 (53.2)	267 (45.4)	–
Duration in ICU	–	123.4 ± 399.4	–
*POD (no., %)*			< 0.001
Yes	36 (11.5)	113 (42.3)	
No	277 (88.5)	154 (57.7)	
*Surgical discipline (no., %)*			< 0.001
Cardiac surgery	4 (1.3)	148 (55.4)	
Thoracic surgery	6 (1.9)	8 (3.0)	
Abdominal surgery	31 (9.9)	31 (11.6)	
Vascular surgery	8 (2.6)	14 (5.2)	
Orthopedic surgery	183 (58.5)	36 (13.5)	
Others	81 (25.9)	30 (11.2)	
*Surgical risk (no., %)*			< 0.001
Low	75 (24.0)	8 (3 0)	
Intermediate	190 (60.7)	68 (25.5)	
High	48 (15.3)	191 (71.5)	
Surgery duration (min.)	140.9 ± 77.6	290.1 ± 114.9	< 0.001
Red blood cell transfusion (ml)	51.1 ± 277.4	856.0 ± 2136.6	< 0.001
Ventilation time (h)	3.6 ± 1.5	26.0 ± 95.7	< 0.001
LOS in hospital (days)	15.4 ± 20.6	24.0 ± 30.3	< 0.001

Data are mean ( ±) unless stated otherwise

*POD* postoperative delirium, *LOS* length of stay, *ICU* Intensive Care Unit

### Influence of POD on LOS in ICU and in hospital

Linear regression results confirm POD as an independent predictor of LOS in the ICU after risk adjustment with perioperative variables (Table 3). Following our model, patients with POD have a 36% (95% CI 4–78%; *p* < 0.001) increase in LOS in ICU independently from their perioperative risk factors. A sensitivity analysis fitting the same regression model without the variable red blood cell transfusion (which contains *n* = 66 missing values) led to very similar results (43% increase; 95% CI 10–86%; *p* < 0.001). Furthermore, the linear regression model confirms that patients with a POD have a 22% (95% CI 4–43%; *p* < 0.001) increase in LOS in hospital after adjusting with perioperative variables. Again, the sensitivity analysis without adjusting for red blood cell transfusion supports this (25% increase; 95% CI 8–45%; *p* < 0.001).

**Table 3:** POD as an independent predictor for LOS in ICU and in hospital: effects were adjusted for perioperative risk factors via a multi-variable linear regression model and are presented as an increase in percent

	POD (adj. effect)	95% CI	*p* value
LOS in ICU (h)	1.36	1.04–1.78	< 0.001
LOS in hospital (days)	1.22	1.04–1.43	< 0.001

POD effect on LOS ICU adjusted for perioperative risk factors (preoperative age, ASA-, NYHA-, MET-, rCRI-classification levels, hearing impairment, history of delirium, number of medication and intra-/postoperative surgical risk, surgical discipline, duration of surgery, red cell blood transfusion, ventilation time). POD effect on LOS in hospital adjusted for perioperative risk factors (such as for the regression analysis for LOS ICU without ventilation time)

*POD* postoperative delirium, *CI* confidence interval, *ICU*  Intensive Care Unit, *LOS* length of stay

## Discussion

### POD incidence and predictors

The POD incidence in this subgroup analyses with patients aged 70 and older was 25.9%. Several preoperative variables showed a significant difference between the POD and non-POD groups. As confirmed in the literature, there was a significant difference in preoperative cognitive testing with the MoCA and positive POD assessment [3–5, 33, 34]. In addition, further studies have shown that cognitive impairment may also have an impact on prolonged hospital stay [35]. However, cognitive impairment as a major preoperative risk marker for POD has been strongly described in systematic reviews as well as in the ESAIC Guideline. Based on the preoperative anesthetic classifications (ASA, NYHA, MET, rCRI), the patients who developed POD were also classified with more pre-existing clinically relevant conditions. Furthermore, the preoperative assessed surgical risk was on average higher in the POD group than in the non-POD group. The ESAIC guidelines recommend, based on their systematic analysis of the study evidence, that ASA classification should be considered a pre-operative risk marker for POD. Furthermore, it is recommended that the factor of surgical risks should also be considered in the risk analysis for POD. These findings are congruent with the POD risk factors described in literature and guidelines [5, 36]. Contrary to what has been reported in the literature, the POD patients in this subgroup did not show significant differences in hearing impairment and a prior POD experience, relative to the non-POD group [37].

POD is a multifactorial complication in which both preoperative predisposing factors as well as intraoperative and postoperative precipitating factors contribute to its development. Significant contributors to the development of POD are the duration time of surgery and the period of ventilation. Patients who developed POD showed a significantly longer operation time of 78 min on average, a longer ventilation time of 25 h and a longer stay in the intensive care unit of 138 h in this subgroup analysis. It should be noted here that outliers, especially in the POD group, influence the time values of ventilation duration and intensive care stay.

### Relationship between ICU stay and POD development

The literature describes, in particular, the large influence of the ICU stays on POD. Due to this significant influence of the ICU stay, this cohort of the subgroup analyzed here was considered in more detail. According to the existing results in the literature, the patients with a subsequent ICU stay had a significantly longer operation time of 149 min more on average, a significantly larger amount of blood transfusion of 805 ml, and a longer ventilation time of 22 h. As mentioned above again a few outliers, especially in the ICU group, characterize the values for ventilation duration and transfusion volume. Furthermore, the results showed that the cohort of patients with an ICU stay also developed POD significantly more often than the opposite group. These results also confirm the findings of other studies that patients with an ICU stay are much more likely to develop POD [38–41]. Another observational study also looked at the occurrence of POD in the ICU and found that POD monitoring alone improved patient outcome [42].

### Impact of POD on LOS in ICU and in hospital

There is various evidence in the literature that patients with a POD or ICU stay have a longer LOS in the hospital [3, 18, 19]. In this regard, we wanted to use this subgroup analysis to show more precisely whether the total length of hospital stay was influenced more by the fact of a necessary ICU stay or primarily by the secondary diagnosis of POD. Patients who developed POD had an average longer hospital stay of about 8 days. However, patients with an ICU stay had a longer average hospital stay of 9 days compared to patients without an ICU stay.

To test whether the occurrence of POD influences LOS in the ICU and in the hospital, a linear regression model was performed, risk-adjusted for perioperative risk factors in both cases. The results of our model showed that the development of POD resulted in a 36% increase in LOS in the ICU independent of perioperative risk factors. Furthermore, the results confirm that patients with POD had a 22% increase in-hospital LOS after risk adjustment. Confirming our findings, another study also found that POD is a robust predictor of LOS in ICU and also has a significant impact on the morbidity and mortality of patients undergoing surgery [43]. From these results, it can be concluded that POD has an independent impact on LOS in ICU and in hospital. An intervention study addressed the problem of POD and prolonged ICU stay and found that a more extended ICU visit model can reduce both POD incidence and LOS [44]. Through the results of our analyses and the supporting findings of the literature, the importance of POD issues for elderly patients overall and specifically for ICU patients is demonstrated. Based on the known risk factors for POD and prolonged ICU stay, risk screening and interventions for prevention need to be further explored and applied in routine clinical practice.

### Limitations

This study has several limitations. A limitation is that the positive delirium diagnosis is based on the results of the delirium tests and not on a diagnosis by a psychologist. Another limitation to be mentioned is that although the regression analysis has included certain risk factors for postoperative delirium, there may be other unobserved confounders. In addition, it has to be considered that the analysis carried out here is a subgroup that exclusively observes patients over 70 years of age.

## Conclusions

The subgroup analysis presented here shows that POD has an independent and significant impact on LOS in ICU and in hospital. The occurrence of POD resulting in a prolongation of the inpatient stay could lead to an increased risk for further postoperative complications for the patient. Furthermore, the already limited resources regarding the availability of ICU beds and the workload of the clinic personnel are very much burdened by a prolonged length of stay. To avoid the scarceness of hospital resources it is of major importance to detect patients at risk for POD by adequate risk screening, so standardized screening in hospitals is necessary.
